# Inter- and Intra-Patient Heterogeneity of Response and Progression to Targeted Therapy in Metastatic Melanoma

**DOI:** 10.1371/journal.pone.0085004

**Published:** 2014-01-06

**Authors:** Alexander M. Menzies, Lauren E. Haydu, Matteo S. Carlino, Mary W. F. Azer, Peter J. A. Carr, Richard F. Kefford, Georgina V. Long

**Affiliations:** 1 Melanoma Institute Australia, Sydney, Australia; 2 The University of Sydney, Sydney, Australia; 3 Westmead Hospital, Crown Princess Mary Cancer Centre, Sydney, Australia; 4 Westmead Hospital, Department of Radiology, Sydney, Australia; 5 Westmead Institute for Cancer Research, Westmead, Australia; The Moffitt Cancer Center & Research Institute, United States of America

## Abstract

**Background:**

MAPK inhibitors (MAPKi) are active in *BRAF*-mutant metastatic melanoma patients, but the extent of response and progression-free survival (PFS) is variable, and complete responses are rare. We sought to examine the patterns of response and progression in patients treated with targeted therapy.

**Methods:**

MAPKi-naïve patients treated with combined dabrafenib and trametinib had all metastases ≥5 mm (lymph nodes ≥15 mm in short axis) visible on computed tomography measured at baseline and throughout treatment.

**Results:**

24 patients had 135 measured metastases (median 4.5/patient, median diameter 16 mm). Time to best response (median 5.5 mo, range 1.7–20.1 mo), and the degree of best response (median −70%, range +9 to −100%) varied amongst patients. 17% of patients achieved complete response (CR), whereas 53% of metastases underwent CR, including 42% ≥10 mm. Metastases that underwent CR were smaller than non-CR metastases (median 11 vs 20 mm, *P*<0.001). PFS was variable among patients (median 8.2 mo, range 2.6–18.3 mo), and 50% of patients had disease progression in new metastases only. Only 1% (1/71) of CR-metastases subsequently progressed. Twelve-month overall survival was poorer in those with a more heterogeneous initial response to therapy than less heterogeneous (67% vs 93%, *P = *0.009).

**Conclusion:**

Melanoma response and progression with MAPKi displays marked inter- and intra-patient heterogeneity. Most metastases undergo complete response, yet only a small proportion of patients achieve an overall complete response. Similarly, disease progression often occurs only in a subset of the tumor burden, and often in new metastases alone. Clinical heterogeneity, likely reflecting molecular heterogeneity, remains a barrier to the effective treatment of melanoma patients.

## Introduction

Molecular heterogeneity exists in all cancers [1], [2], particularly melanoma [3]–[5]. Genetic divergence occurs during clonal evolution, resulting in inter- and intra-tumoral molecular heterogeneity within patients [3], [6], [7]. Certain driver genetic aberrations exist in all tumor cells within an individual, but several others exist in subclones, conferring varying degrees of drug resistance [2]. Intrinsic resistance mechanisms present in subclones of the overall tumor burden diminish the initial response to systemic treatment, and these and acquired mechanisms result in disease progression. Ultimately the presence or development of these mechanisms influence the initial response to systemic treatment, time to progression, and overall survival. The influence and heterogeneity of the tumor micro-environment is also increasingly understood to play a role in tumor cell heterogeneity and treatment outcome [8].

Clinically, inter- and intra-patient molecular heterogeneity is manifest by the variable responses observed between and within patients treated with targeted therapies. BRAF inhibitors, used as single agents or in combination with MEK inhibitors, are active in most patients with metastatic melanoma, but the extent of response and time to progression are variable between patients, and complete responses are uncommon [9]–[11]. Patterns of disease progression are also variable, with existing metastases progressing or new metastases developing at the same time as ongoing response in other metastases [12], [13]. The terms “mixed response” and “isolated progression” are now used commonly, however these terms have not yet been accurately defined, and there is little known as to the prevalence or predictors of these phenomena, nor the clinical outcomes of patients with these patterns of response and progression.

We therefore sought to examine the patterns of response and progression to targeted therapy by measuring every metastasis ≥5 mm via computed tomography (CT) in a cohort of patients with metastatic melanoma treated with combined BRAF and MEK inhibitors.

## Patients and Methods

### Patients and Treatment

All MAPK inhibitor naïve BRAF-mutant metastatic melanoma patients treated with dabrafenib and trametinib (CombiDT) on parts B–D of the BRF113220 Phase 1/2 [11] trial (NCT01072175) at Westmead Hospital in association with Melanoma Institute Australia were included for analysis. The collection and analysis of clinical data was approved by the Westmead and Royal Prince Alfred Hospitals Human Research Ethics Committees (Protocol No. X11-0023 and HREC/11/RPAH/32) and written informed consent was obtained from each patient. Patients received a range of doses of dabrafenib and trametinib. Patient demographic and disease characteristic data at trial entry were collected.

### Disease Assessments

CT scans of 3 mm slice thickness were performed at baseline and then every 8 weeks as per the clinical trial protocol. In addition to the RECIST v1.1 assessments [14] conducted prospectively as part of the clinical trial, a more detailed radiologic assessment of every metastasis ≥5 mm diameter in long axis (lymph nodes ≥15 mm in short axis) visible on CT was performed on every scan. This was referred to as the “ALL metastasis” assessment, and was conducted retrospectively, blinded to the RECIST assessment and clinical data. Measurements were made on each scan to the nearest millimeter using the IntelePACS© computer software program.

RECIST data were used only as a comparison to the ALL metastasis assessment data to assess for concordance of these measures for best overall response, time to best response (TTBR), and progression-free survival (PFS) (see supplementary methods).

The patient’s overall response at each time point was determined using similar criteria as RECIST [14], but included all metastases ≥5 mm to calculate the sum of diameters (SoD). Disease progression was defined as the development of new metastases and/or a ≥20% and ≥5 mm increase in the sum of diameters of all metastases from nadir.

In addition, a response was recorded for each individual measured metastasis at each time point and classified as complete response (CR, disappearance or to less than 10 mm for a lymph node), partial response (PR, ≥30% reduction), stable disease (SD, neither CR/PR/PD) or progressive disease (PD, ≥5 mm and ≥20% growth).

At first radiologic assessment, for this study, a uniform response was predefined as ≥80% of metastases having a complete or partial response with no progressing or new metastases. A mixed response was defined as <80% of metastases having a complete or partial response, or the presence of any progressing or new metastases.

### Statistical Analysis

Patient demographic and clinical features were tested for association with uniform versus mixed response at first scan using the Fisher’s Exact Test, Pearson’s χ^2^, and/or the Mann Whitney U test as appropriate. Overall survival (OS) and PFS were calculated from the date of commencement of targeted therapies to the date of last follow-up or date of progression, respectively. Univariate time to event analyses were conducted with the Kaplan-Meier method together with the Log Rank test for comparison of categorical covariates, and with the Cox proportional hazards method for continuous covariates. Multivariate overall survival was conducted with the Cox proportional hazards method. When comparing the two assessment methods (RECIST and ALL metastasis), best overall response was deemed concordant if there was ≤10% difference in the percentage degree of best response and also within the same response category. Time to best response and progression-free survival were concordant if they occurred at the same time (on the same scan) by both measures. All statistical analyses were conducted with IBM SPSS Statistic v21.

## Results

### Patient Demographics and Disease Characteristics

Twenty-four patients were included for analysis. The patient population was typical for patients with BRAF-mutant metastatic melanoma; the median age of patients was 51 years, 54% of patients were men, 85% of patients had the V600E genotype, and 58% of patients had stage M1c melanoma (Table 1). All patients were MAPK inhibitor naïve. Although several dosing regimens were administered, 71% of patients were treated with trametinib at the recommended part two dose of 2 mg daily in combination with dabrafenib from trial commencement (Table 1). Two patients received dabrafenib monotherapy until disease progression, after which 2 mg daily trametinib was added.

**Table 1:** Patient demographics and clinical characteristics.

Feature	All patients		Uniform Responseat First Scan*		Mixed Responseat First Scan*		*P*-value#
	N	%	N	%	N	%	
**Number of patients**	24	100	15	62	9	38	–
**Age (years)**							
Median	51	–	57	–	42	–	0.290
Range	29–78	–	28–77	–	38–74	–	
**Sex**							
Male	13	54	8	53	5	56	0.625
Female	11	46	7	47	4	44	
**BRAF genotype**							
V600E	20	85	13	87	7	78	0.486
V600K	4	15	2	13	2	22	
**ECOG PS**							
0	19	79	11	73	8	89	0.360
1	5	21	4	27	1	11	
**AJCC Stage**							
M1a	5	21	3	20	2	22	0.418∧
M1b	5	21	4	27	1	11	
M1c	14	58	8	53	6	67	
**Baseline LDH**							
<1×ULN	19	79	13	87	6	67	0.255
>1×ULN	5	21	2	13	3	33	
**Drug doses (Dab/Tra)**							
300/2	8	33	6	40	2	22	Not Tested
300/1.5	1	4	0	0	1	11	
300/1	4	17	2	13	2	22	
300/0 then 300/2 at PD	2	8	2	13	0	0	
150∼/2	8	33	4	27	4	44	
300∼/2	1	4	1	7	0	0	
Dab with 2 mg Tra	17	71	11	73	6	67	0.539
Dab with <2 mg Tra	7	29	4	27	3	33	

Abbreviations: ECOG PS, Eastern Cooperative Oncology Group Performance Status; AJCC, American Joint Committee on Cancer; LDH, lactate dehydrogenase; ULN, upper limit of normal; Dab, dabrafenib total daily dose; Tra, trametinib daily dose; PD, progressive disease.

hydroxymethylcellulose dabrafenib preparation.

testing M1a & M1b versus M1c.

Uniform response: ≥80% of metastases with a complete or partial response and no progressing or new metastases. Mixed response: <80% of metastases with a complete or partial response, or the presence of any progressing or new metastases.

^#^ testing uniform versus mixed response cohorts.

### Baseline Disease Assessments

135 metastases from the 24 patients were included for assessment (median 4.5 per patient, range 1–18), substantially more than included as RECIST targets (*N = *56, median 2 per patient, range 1–5) (Table 2). The median diameter of metastases was the same as RECIST targets (16 mm), but ranged from a minimum 5 mm rather than 10 mm. Seventy-six percent (*N = *102) of metastases were ≥10 mm, and 46 (45%) of these had not been included as RECIST targets. Most frequent sites of disease included lung and subcutaneous/soft tissue (SQ) (36% and 32% respectively) (Table 2).

**Table 2:** Baseline disease assessments by examining RECIST targets versus ALL metastases.

	RECIST targets	ALL metastases
Total	56	135
**Diameter (mm)**		
Median	16	16
Range	10–108	5–108
Number ≥10 mm	56	102
**Number per patient**		
Median	2	4.5
Range	1–5	1–18
**Sum of Diameters (mm)**		
Median	48	100
Range	10–174	11–317
**Site of metastases (n, %)**		
SQ	13, 23%	43, 32%
Lymph node	10, 18%	15, 11%
Lung	16, 29%	48, 36%
Liver	12, 21%	24, 18%
Gastrointestinal*	5, 9%	5, 4%

Abbreviations: SQ, subcutaneous and soft tissue.

Gastrointestinal sites include adrenal (*N = *3), small bowel (*N* = 1), pancreas (*N* = 1).

### Overall Patient Response

**Figure 1:**
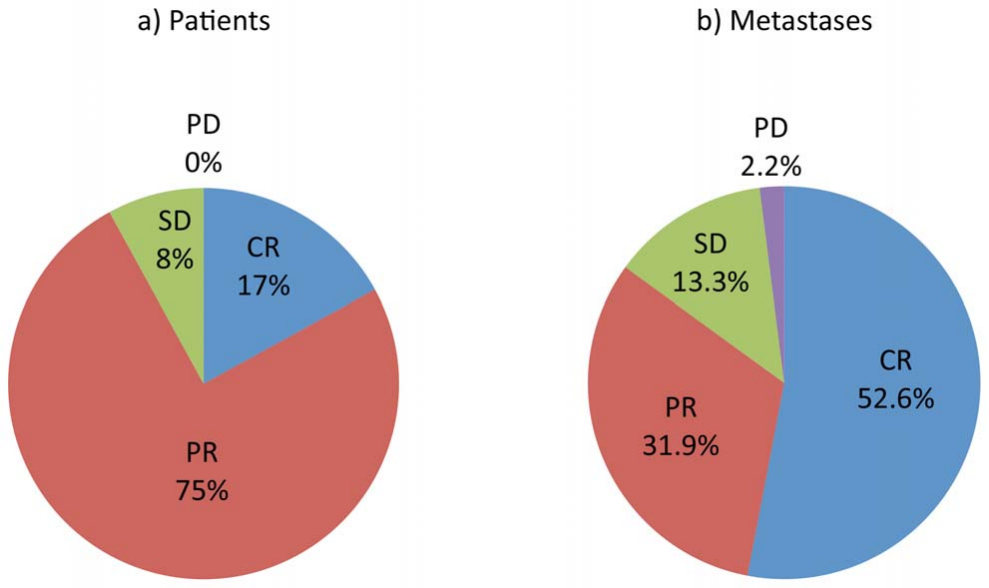
The proportions of categories of response a) by patients (*N* = 24), b) by metastases (*N* = 135). Abbreviations: CR, complete response; PR, partial response; SD, stable disease; PD, progressive disease.

**Figure 2:**
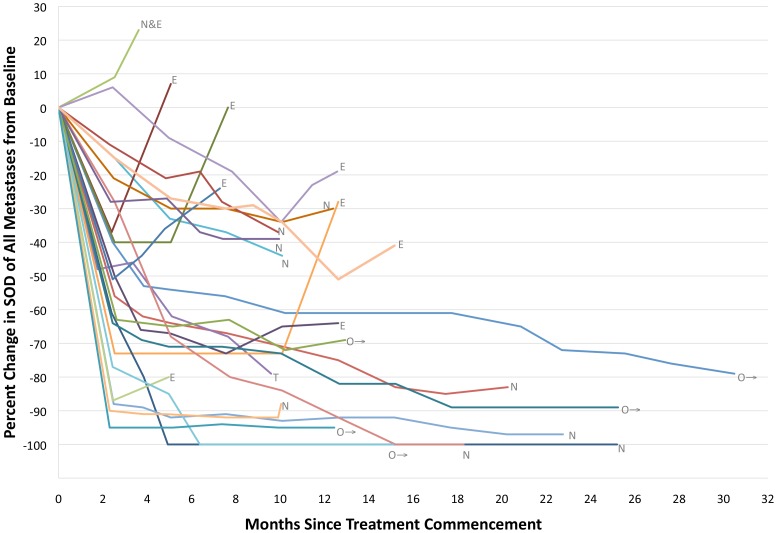
Inter-patient heterogeneneity of response and progression with CombiDT. Plot of the percent change in the sum of diameters of all metastases ≥5 mm within an individual patient compared to baseline at various time points during treatment with CombiDT until disease progression. Each line represents an individual patient. Abbreviations: E, disease progressing due to existing lesions; N, new lesions; N+E, new and existing lesions; O→, ongoing response without progression; T, treatment ceased due to toxicity.

**Figure 3:**
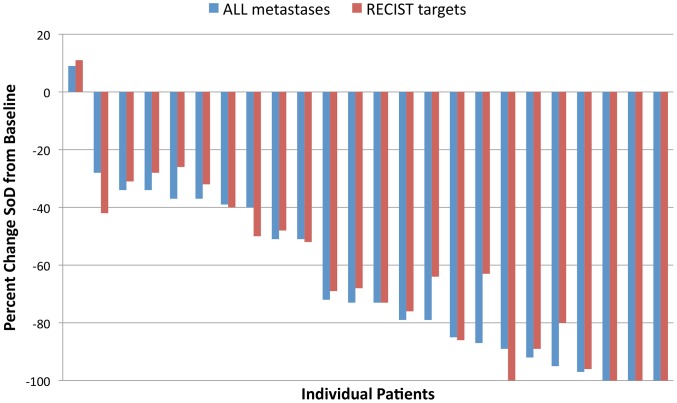
The degree of overall best response for each patient by RECIST and ALL metastasis disease assessments.

The majority of patients had a response to treatment. When all metastases ≥5 mm were measured, 17% (*N* = 4) of patients had a complete response and 75% (*N* = 18) had a partial response to treatment (Figure 1a). No patients had progressive disease as best response. The median time to best response was diverse (median 5.5 months, range 1.7 to 20.1 months), and there was variability in the degree of response at first assessment (median change −49%, range +9 to −95%), the kinetics of response (% change over time), and the degree of best response (median change −70%, range +9 to −100) within the patient population (Figure 2). The degree of best overall response by ALL metastasis and RECIST assessment measures was concordant in 19/24 (79%) patients (Figure 3), the category of response was concordant in 20/24 (83%) patients, and TTBR was concordant in 17/24 (71%) patients.

### Individual Metastasis Response

**Figure 4:**
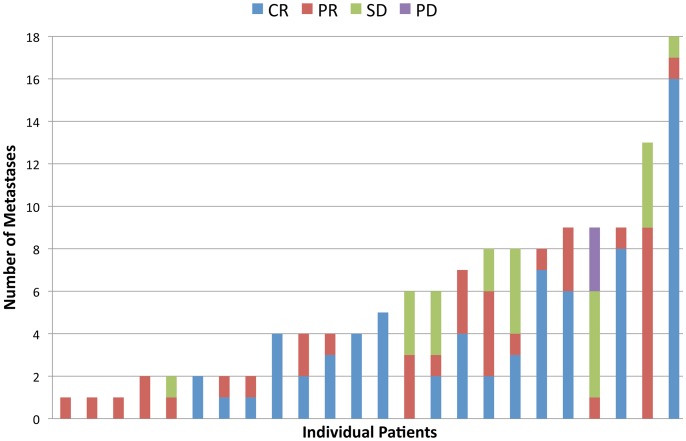
The best response of each individual metastasis within each patient. Abbrevations: CR, complete response; PR, partial response; SD, stable disease; PD, progressive disease.

Ninety-three percent (126/135) of metastases had some reduction in size with treatment and 84.5% (114/135) had either a complete or partial response. Only 2.2% (3/135) of metastases demonstrated progressive disease at first assessment, all within the same patient. Importantly, 52.6% (*N* = 71) of metastases had a complete response (Figure 1b, Figure 4). Of 102 metastases ≥10 mm diameter, 42% (43/102) had a complete response, and 41% (23/56) RECIST target metastases had complete response.

**Figure 5:**
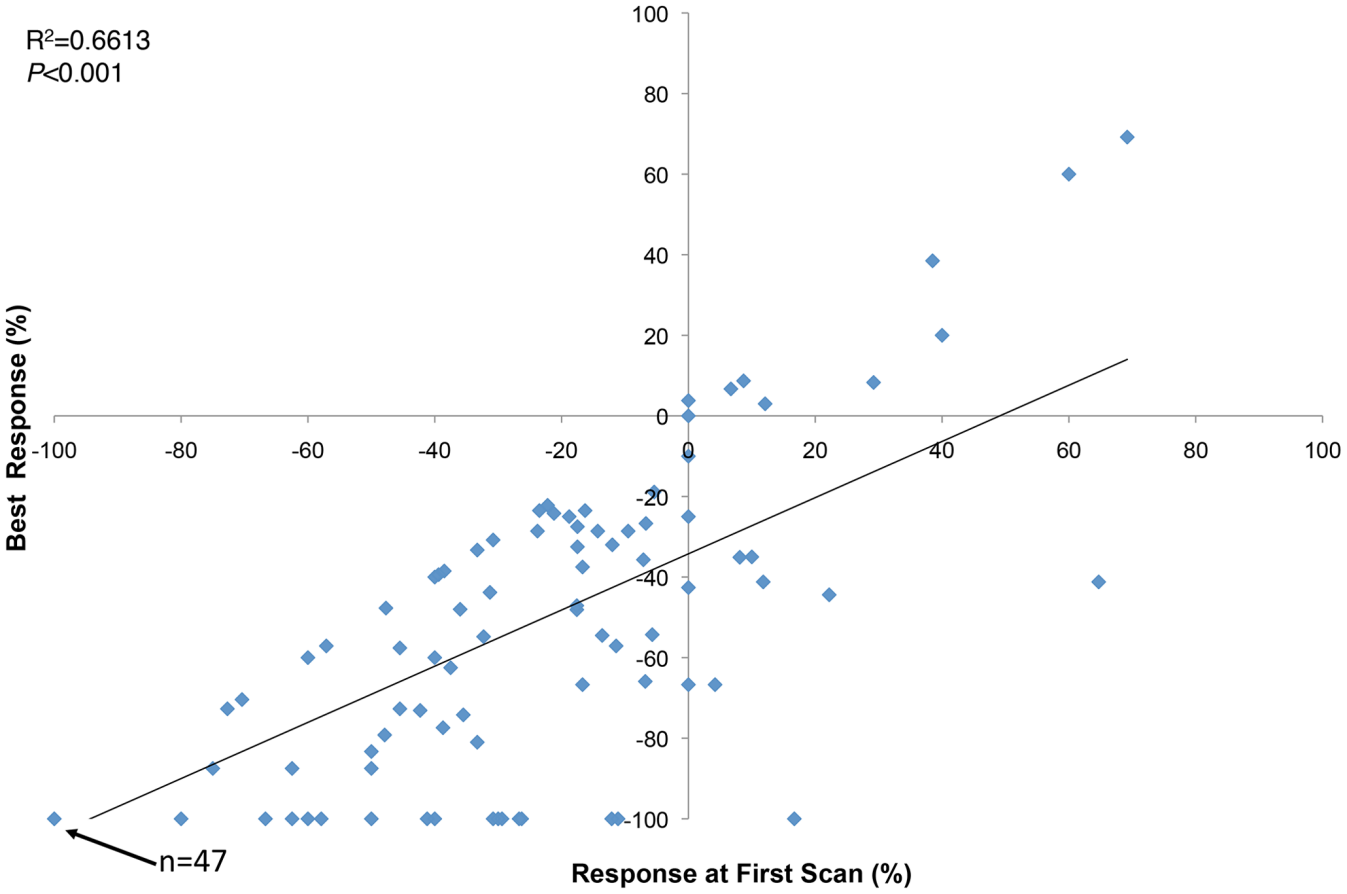
Correlation of the response of individual metastases at first scan versus best response (*N* = 135).

The median TTBR for all metastases was 12.1 weeks (range 7.3–87.6 weeks) (Table 3). Compared with subcutaneous and soft tissue metastases (median 8.3 weeks), median TTBR was significantly longer for lymph nodes (30.3 weeks, *P = *0.009) and liver metastases (31.7 weeks, *P* = 0.038), but not significantly different for lung metastases (8.0 weeks, *P* = 0.076). TTBR was significantly shorter as metastases decreased in size (HR = 0.98, 95% CI 0.96–0.998, *P* = 0.030), and the degree of response at first scan correlated with the degree of best response (R^2^ = 0.6613, p<0.001) (Figure 5).

**Table 3:** Factors influencing individual metastasis response to treament; time to best response by metastasis site, and the effect of metastasis site and size on response.

Site ofmetastasis	Median Time to BestResponse (Range) Weeks	CR		PR/SD/PD		*P*-value*
		*N*	Median Size(Range) mm	*N*	Median Size(Range) mm	
**All**	12.1 (7.3–87.6)	71	11 (5–44)	64	20 (5–108)	<0.001
**SQ**	8.3 (7.6–56.3)	24	10 (7–30)	19	20 (10–98)	<0.001
**LN**	30.3 (7.7–87.6)	7	22 (15–31)	8	21 (17–48)	0.38
**Lung**	8.0 (7.3–63.9)	27	9 (5–44)	21	15 (5–44)	0.036
**Liver**	31.7 (7.7–56.0)	10	18 (7–27)	14	31 (16–47)	0.006

*P*-value for comparison of median size of lesions with CR versus non-CR, Mann Whitney U test.

Abbreviations: CR, complete response; PR, partial response; SD, stable disease; PD, progressive disease; SQ, subcutaneous and soft tissue; LN, lymph node.

There was no significant difference in the rate of complete response by disease site (*P*>0.05) (Table 3). Metastases that had a complete response were significantly smaller compared with metastases that had PD/SD/PR (median 11 mm vs 20 mm, *P*<0.001). This factor remained significant when stratifying by disease sites for lung, liver, and SQ metastases (all *P*<0.05), but not for lymph nodes (*N* = 15).

**Figure 6:**
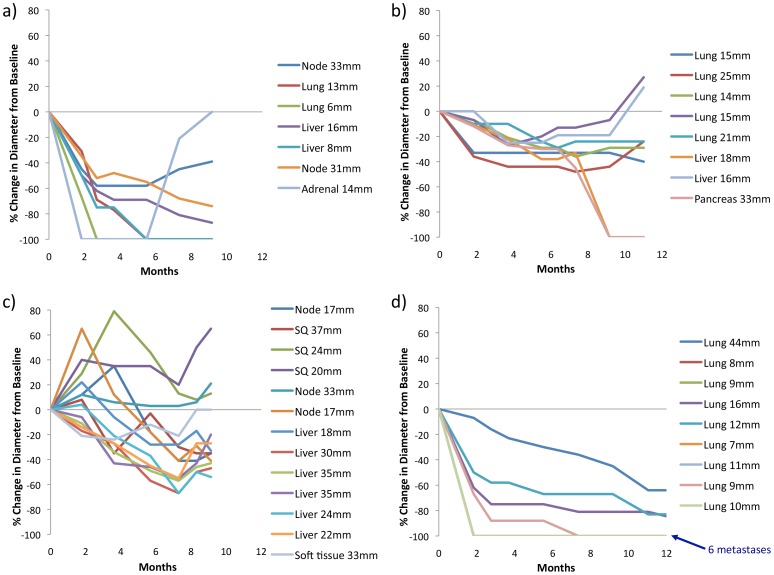
Intra-patient heterogeneneity of response and progression with CombiDT. Example plots of the percent change in the diameter of individual metastases within four patients (a-d) compared to baseline at various time points during treatment until overall disease progression. The degree and kinetics of response of individual metastases vary within a patient. Similarly, progression often occurs only in a subset of the overall tumour burden. Patient D had disease progression in new lesions only.

Plots of the response of individual metastases over time within individual patients (Figure 6) demonstrated the marked variability in the degree of first response and best response, the kinetics of response, and the time to best response for each individual metastasis.

Sixty-two percent (15/24) of patients had a uniform response at first assessment, and 38% (9/24) of patients had a mixed response. Patient demographics, disease characteristics and CombiDT doses received were similar in the two groups (Table 1). The two patients that received dabrafenib monotherapy until disease progression had a uniform response to treatment.

### Patterns of Disease Progression

**Figure 7:**
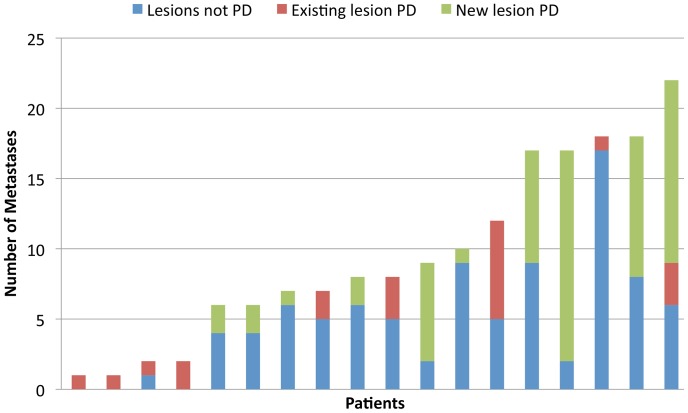
Intra-patient heterogeneity of disease progression. The number and type of metastases progressing at time of disease progression.

At the time of analysis 18 (75%) patients had disease progression (PD) (Figure 2). Median PFS was 8.2 months (range 2.6 to 18.3 months). PFS was highly concordant by ALL metastasis and RECIST assessment methods (14/18, 77% of patients). Fifty percent of patients progressed in new metastases only, 44% in existing metastases only, and 6% in both new and existing metastases simultaneously. There was no dominant site of disease progression, but four (22%) patients with no prior history of brain metastases progressed in new metastases in the brain. At time of PD, the median proportion of metastases progressing in an individual compared to the total tumor burden ever (including all metastases at baseline and new metastases) was 49% (range 6 to 100%) (Figure 7). Only one metastasis that underwent complete response subsequently progressed (1.4%, 1/71).

### Survival Analyses

The burden of disease at baseline (SoD of ALL metastasis) and the degree of overall response at first scan did not correlate with PFS, 12-month survival or OS (Table 4). The type of initial response (uniform versus mixed) similarly did not correlate with PFS. 12-month and OS, however was significantly inferior for mixed responders (67% and median 14.2 months) compared with uniform responders (93% and median not reached, *P* = 0.009), a result which remained significant when adjusting for baseline disease burden (HR = 5.1, 95% CI: 1.2–21.1, *P* = 0.025).

**Table 4:** Univariate progression-free and overall survival.

Outcome	Factor	*P*-value
PFS	Baseline SoD	0.101
	Percent Response at First Scan	0.084
	Uniform versus Mixed Response at First Scan*	0.124
OS	Baseline SoD	0.349
	Percent Response at First Scan	0.105
	Uniform versus Mixed Response at First Scan*	0.009

Abbreviations: SoD, sum of diameters.

Uniform response: ≥80% of metastases with a complete or partial response and no progressing or new metastases. Mixed response: <80% of metastases with a complete or partial response, or the presence of any progressing or new metastases.

## Discussion

This is the first systematic study of patterns of clinical response and progression to MAPK targeted therapy in all assessable individual metastases in patients with metastatic melanoma, demonstrating that melanoma response and progression is heterogeneous between and within patients. Most individual metastases undergo a complete response to treatment, yet only a small proportion of patients achieve an overall complete response. Disease progression is similarly heterogeneous, both in timing and nature. Many patients have disease progression in a subset of their overall tumor burden, and often in new metastases only. Metastases that initially undergo complete response with treatment seldom subsequently progress, and a more heterogeneous initial response to treatment is associated with shorter overall survival.

Results of this study are strengthened by the detailed clinical assessment of patients on the most highly active targeted therapy in melanoma [11], the use of a standard modality for disease assessment (3 mm slice thickness CT) at predetermined specified time points, inclusion of every metastasis visible and measurable on CT scan (≥5 mm or ≥15 for lymph nodes), and an assessment of every individual metastasis across every time point from baseline prior to treatment until disease progression. The inclusion of all metastases as targets for assessment, as opposed to the maximum 5 target metastases for RECIST (and maximum 2 in any one organ) provided a more detailed assessment, with increased ability to assess for intra-patient heterogeneity. This question has been previously addressed in studies of ^18^F-labelled fluorodeoxyglucose positron emission tomography (PET) metabolic response to single agent BRAF inhibitors at day 15, with varying results, one study examining 5 target metastases and observing a homogeneous response [15], while the other examined every metastasis and observed heterogeneity [16].

In this study, most metastases achieved a complete response with treatment. These metastases were located at any body site, and tended to be smaller than those that did not undergo complete response, however, some metastases several centimeters in diameter still had complete response. The reasons why smaller metastases have a higher complete response rate may be because they have to shrink less to become clinically occult, however, the observation that these metastases seldom subsequently progress perhaps supports alternative hypotheses, for example, they undergo a more effective secondary immune response [17], [18], or contain less molecular or microenvironmental heterogeneity, with less resistant tumor subclones. This observation warrants further research, particularly as larger metastases may be amenable for resection prior to therapy, and adjuvant trials for occult metastatic disease are in progress.

Despite heterogeneity observed in the degree and timing of best overall response amongst patients, most metastases undergo the majority of tumor shrinkage by 3 months of treatment. Metastases that have not undergone meaningful initial clinical response (e.g. persisting local symptoms) by 3 months may therefore warrant treatment with local therapy (surgery, radiotherapy). Furthermore, in selected patients where the vast majority of metastases have undergone complete response, remaining metastases could be treated locally to render the patient free of overt disease. The observation that the majority of tumor response occurs early during treatment also suggests that additional systemic therapies (e.g. immunotherapy) should be incorporated early in the course of MAPK inhibitor treatment. Translational data demonstrating early immune cell infiltration into tumors soon after treatment commencement (as early as day 3) further supports this, and may indicate that immunotherapies should be combined from the start of MAPK inhibitor treatment [17], [18].

Disease progression occurred at varying time points among the patient cohort, and there was a high rate of disease progression due to the emergence of new metastases. Often, patients progressed in only a few metastases, with the remainder of disease under treatment control. In this instance, disease progression may therefore not equate to overt treatment failure, and local treatment (e.g. surgery, radiotherapy) may be delivered to progressing metastases with systemic treatment continued for ongoing clinical benefit to the remainder of drug-sensitive disease [12], [13], 19. This approach may be more beneficial than a switch to immunotherapy (e.g. ipilimumab), as little efficacy has been observed in this setting [20], [21], likely at least in part due to the release of MAPK inhibition, whereas relative ongoing MAPK inhibition still occurs in resistant tumors with continued MAPK inhibitor treatment [22].

In this study cohort, a mixed response at first assessment correlated with shorter overall survival, but not progression-free survival. This result was likely influenced by small number of patients, the doses of therapy received, and the fact that many patients progressed in new metastases alone. Subsequent treatments may have also influenced overall survival. Despite this, however, this finding warrants validation in future studies, as biomarkers to predict treatment outcome are scant, and the method of categorizing response in this study could be reproduced without additional procedures such as PET.

The clinical heterogeneity of tumor response and progression demonstrated in this study likely reflects underlying molecular heterogeneity. The majority of the melanoma burden in patients is sensitive to MAPK inhibition, however, a varying proportion of primarily resistant subclones exist at baseline, and resistance may also be acquired during treatment. This heterogeneity complicates clinical management, confounds biopsy driven biomarker research, and remains a barrier to the effective treatment of melanoma patients, including the deployment of biopsy-driven adaptive clinical trial design. A broader multi-targeted treatment approach from the outset (e.g, MAPK and PI3K inhibitors) may improve response rates and prolong survival, but will likely face the same problem of clonal drug resistance and treatment failure. Combinations of MAPK inhibitors and novel immunotherapies (e.g. PD-1 antibodies) may provide more complete and durable responses.
